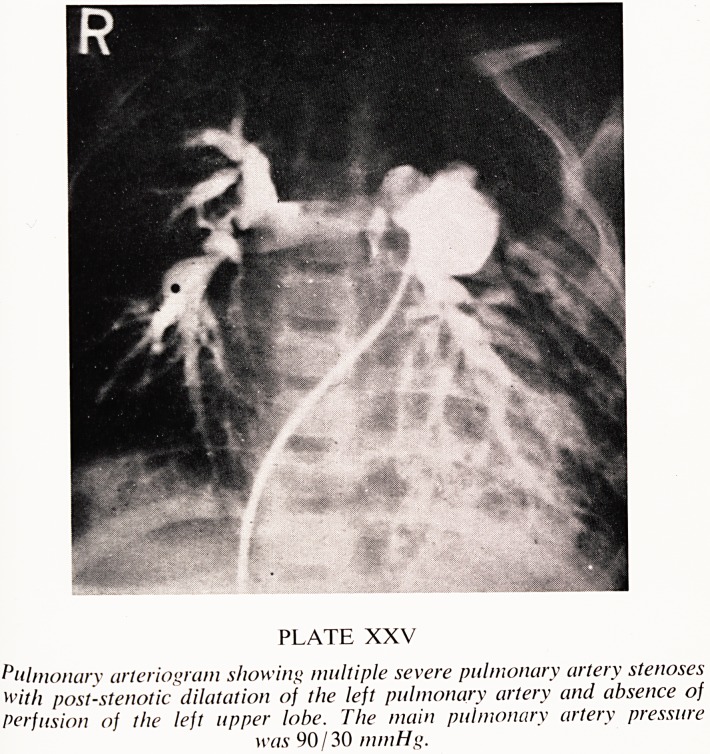# Cardiac Lesions in Children with "Hypercalcaemic" Facies

**Published:** 1969-10

**Authors:** S. C. Jordan


### CARDIAC LESIONS IN CHILDREN WITH "HYPERCALCAEMIC" FACIES

**S. C. Jordan**

The factors causing congenital heart disease, apart from the well known effects of rubella in the mother during the first three months of pregnancy, are in general obscure. The association of congenital heart disease with multiple non-cardiac abnormalities may form the basis for an investigation into other aetiological factors. The combination of cardiac defects with infantile hypercalcaemia is a fairly recent discovery; the exact nature of this association is still not understood. In 1956 Schlesinger, Butler, and Black drew attention to the occurrence of a characteristic facial appearance in infants with severe hypercalcaemia, and in 1964 Beuren *et al.* drew attention to the syndrome of supra-aortic stenosis and a similar facial appearance. The connection between these two apparently unrelated syndromes was pointed out by Black and Bonham Carter (1963), who found that patients previously seen with infantile hypercalcaemia were presenting later in life with cardiac defects, particularly aortic stenosis. It has since been suggested that the cardiac defects may be the result of the deposition of calcium during the phase of hypercalcaemia.

### PATIENTS STUDIED

The present study concerns 13 patients with cardiovascular lesions who have the typical facies, and 5 with a less typical facies, without all of the features. An example of a typical facies is shown in Plate XXIII. The age of the patients when seen with these cardiac lesions varied from 3 months to 15 years. They included 2 sibs from one family and first cousins from another family.

An additional 3 patients had a history of congenital heart disease in a first degree relative, but without an abnormal facies. 8 of the patients were considered to be normal mentally, 6 were mildly backward and 3 moderately and 1 severely retarded. 3 children had squints and 3 had had operations for the correction of squint. Several had pigment abnormalities in the retina. 1 patient had epiloia.

### *Hypercalcaemia*

Only 2 of the patients had a clinical history of infantile hypercalcaemia. 3 additional patients had a serum calcium of between 11.9 and 12.7 mg./100 ml., i.e. above the upper limit of normal, but this was not associated with any symptoms, and in 1 patient was corrected by stopping vitamin D supplements. In the remaining patients the serum calcium was normal, and although no figures were available for the first year of life, careful questioning failed to reveal any history suggestive of infantile hypercalcaemia.

### *Cardiac Lesions*

The principle lesions found are shown overleaf.

**Cardiovascular Lesions**

| | |
|---|---|
| Pulmonary artery stenosis | 9 |
| Aortic valve stenosis | 6 |
| Supra-aortic stenosis | 5 |
| Coarctation | 5 |
| Ventricular septal defect | 3 |
| Atrial septal defect | 2 |
| Ductus | 1 |
| Fallot's tetralogy | 1 |
| Subaortic stenosis | 1 |

In all but 2 patients, the diagnosis was confirmed by cardiac catheterisation. The most common abnormalities found were multiple stenoses of the pulmonary arteries (Plates XXIV and XXV), coarctation of the aorta, and aortic or supra-aortic stenosis.

### DISCUSSION

It is clear that certain cardiac lesions occur relatively much more commonly in this group, namely pulmonary artery stenosis and supra-aortic stenosis. Both these conditions have been reported as occurring several times in a single family (Eisenberg *et al.*, 1964), which suggests that these lesions may be genetically determined. The association between infantile hypercalcaemia and the cardiac lesions is still not clear. The suggestion that the lesions are caused by the deposition of calcium in the aorta and pulmonary arteries is not supported by the infrequency of a history of hypercalcaemia in this series, and it seems unlikely that hypercalcaemia sufficient to cause ectopic calcification would occur without other symptoms. A more likely explanation is that hypercalcaemia and the cardiac lesions are both genetically determined, but variable parts of the same syndrome. It is also suggested that there is a syndrome consisting of several abnormalities of which any number may occur in any one individual. In this context it is interesting that McDonald *et al.* (1969) have described the cardiac lesions in patients with normal facies. The various factors involved in the syndrome are thus:—

| | |
|---|---|
| Facial: | Hypertelorism.<br>Epicanthic folds.<br>Squint.<br>Carp mouth.<br>Dental abnormalities. |
| Biochemical: | Hypercalcaemia.<br>Hypercholesterolaemia. |
| Central nervous system: | Mental retardation.<br>Ataxia. |
| Cardiac: | Pulmonary artery stenosis.<br>Supra- aortic or aortic stenosis.<br>Coarctation of aorta.<br>V.S.D., A.S.D., Fallot (less common). |

It seems likely that other conditions with these features may occur without cardiac abnormalities, although these do not seem to have been reported.

PLATE XXIII

*Facies of a 4.5 year old girl with supra-aortic stenosis. I.Q. 62. Note the widespread eyes, epicanthic folds and snub nose. There was no history to suggest infantile hypercalcaemia and calcium studies were normal.*

PLATE XXIV

*Multiple pulmonary artery stenoses in a 2.5 year old boy with hypercalcaemia and severe retardation. There are narrowings at most points of bifurcation, with post-stenotic dilatation.*

PLATE XXV

*Pulmonary arteriogram showing multiple severe pulmonary artery stenoses with post-stenotic dilatation of the left pulmonary artery and absence of perfusion of the left upper lobe. The main pulmonary artery pressure was* 90/30 *mmHg.*

### References

Beuren, A. J., Schulze, C., Eberle, P., Haranjanz D., and Apitz, J. (1964) Amer. J. Cardiol. *13*, 471.

Black, J. A. and Bonham Carter, R. E. (1963) Lancet. *2*, 745.

Eisenberg, R., Young, D., Jacobson, B., and Boit, A. (1964) Amer. J. Dis. Child. *108*, 341.

McDonald, A. H., Gerlis, L. M. and Somerville, J. (1969) Brit. Heart J. *31*, 375.

Schlesinger, B. E., Butler, N. R. and Black, J. A. (1956) Brit. Med. J. *1*, 127.

## Figures and Tables

**PLATE XXIII f1:**
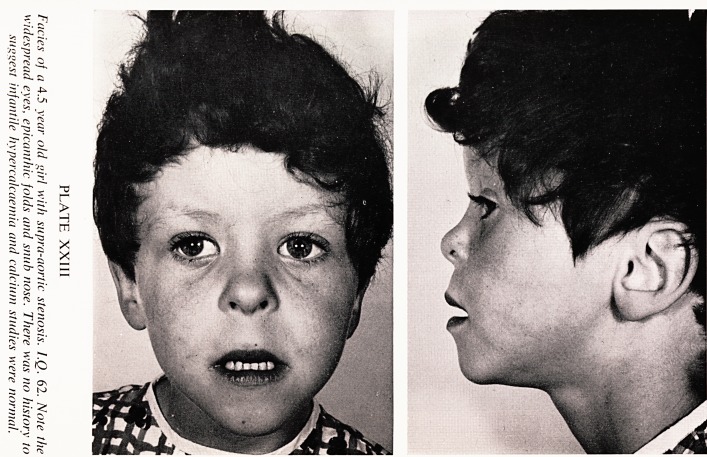


**PLATE XXIV f2:**
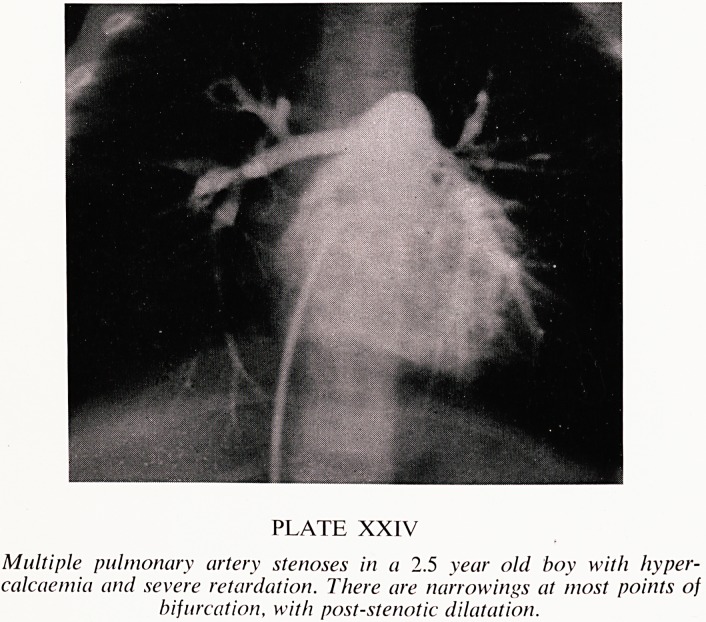


**PLATE XXV f3:**